# Prospective Assessment of Myocardial Deformation in Fetuses With Suspected Aortic Coarctation

**DOI:** 10.1159/fdt/acuag016

**Published:** 2026-10-12

**Authors:** Fleur Zwanenburg, Janine Trigler-Rinner, Renée M. Smit, Renate Oberhoffer, Annette Wacker-Gussmann, Arend D.J. Ten Harkel, Monique C. Haak

**Affiliations:** a Department of Obstetrics and Fetal Medicine, Leiden University Medical Center, Leiden, The Netherlands; b Center for Congenital Heart Disease Amsterdam-Leiden (CAHAL), Leiden, The Netherlands; c Technical University of Munich, TUM School of Medicine and Health, German Heart Center, TUM University Hospital, Pediatric Cardiology and Congenital Heart Defects, Munich, Germany; d Department of Pediatric Cardiology, Leiden University Medical Center, Leiden, The Netherlands

**Keywords:** Aortic coarctation, Fetal heart, Prenatal imaging, Speckle-tracking, Myocardial deformation

## Abstract

**Introduction:**

Prenatal identification of true aortic coarctation (CoA) remains challenging, as evidenced by high false-positive rates. Myocardial deformation has been proposed to improve prediction of postnatal outcome. This prospective study aimed to determine whether fetal myocardial deformation analysis can differentiate true CoA from false-positive cases throughout gestation.

**Methods:**

All fetuses with a prenatal suspicion of isolated CoA were prospectively recruited at our fetal cardiology services. Cases were classified as true CoA or false-positives based on their postnatal diagnosis. Speckle-tracking echocardiography was performed longitudinally to assess left and right ventricular global longitudinal strain (GLS), strain rate, and segmental strain throughout gestation. Measurements were compared between true CoA and false-positive cases.

**Results:**

Thirty-eight fetuses were included (19 true CoA and 19 false-positive cases). True CoA cases exhibited smaller left-sided cardiac dimensions, including the aortic valve (mean Z-score −3.2 vs. −1.6, *p* < 0.001), aortic isthmus (−3.5 vs. −1.7, *p* = 0.001), and mitral valve (−4.9 vs. −3.1, *p* < 0.001), whereas right-sided cardiac dimensions were comparable (pulmonary valve Z-score 3.0 vs. 2.6, *p* = 0.64; arterial duct Z-score 1.9 vs. 3.2, *p* = 0.61). No clinically relevant differences in myocardial deformation parameters were observed between groups, with similar left ventricular GLS (−20.1% vs. −20.5%, *p* = 0.57), right ventricular GLS (−20.0% vs. −19.1%, *p* = 0.13), strain rates, and segmental strain.

**Conclusion:**

Fetal myocardial deformation analysis does not provide additional predictive value for postnatal outcome in prenatally suspected isolated CoA. Conventional assessment of cardiac dimensions and flow patterns remains the cornerstone of prenatal CoA diagnosis, yet current imaging modalities cannot provide sufficient diagnostic certainty to safely triage perinatal management.

## Introduction

Aortic coarctation (CoA) is a localized narrowing of the aorta, typically at the aortic isthmus, and represents 4–6% of all live births affected by congenital heart disease [1]. A prenatal diagnosis is critically important as timely postnatal management with prostaglandins can prevent life-threatening circulatory shock and metabolic acidosis [2, 3]. Prenatal identification of CoA remains, however, challenging and is often regarded as the “Achilles’ heel” of fetal echocardiography. The diagnosis primarily relies on ventricular and great arterial size disproportion, but these ultrasound markers are subject to both high false-negative and high false-positive rates, even in the hands of experts [2, 4–7]. In false-positive cases, the cardiac disproportion typically reflects physiological fetal right-sided dominance, which closely resembles the pathological disproportion as seen in true CoA. Consequently, approximately 40–60% of cases exhibiting left-to-right asymmetry in the prenatal setting are unnecessarily treated as potential coarctation, resulting in unnecessary parental anxiety and unwarranted neonatal intensive care admissions immediately after birth [8, 9].

Notably, the mechanisms underlying prenatal cardiac asymmetry in the development of CoA remain incompletely understood. Hemodynamic alterations have primarily been implicated, with the asymmetry arising either from impaired outflow due to the aortic obstruction – where increased left ventricular afterload reduces ventricular filling and function – and/or from primary limitations in left-heart inflow caused by reduced right-to-left shunting such as restrictive or redundant foramen ovale or persistent left superior vena cava [10–13]. In either case, myocardial loading conditions and contractile performance are thought to be altered, suggesting that intrinsic myocardial remodeling or dysfunction – as seen postnatally long after CoA repair – may already start in utero [14, 15].

Over the past decade, speckle-tracking echocardiography has emerged as a promising modality to assess cardiac function. This technique quantifies myocardial deformation by tracking the motion of defined myocardial regions (kernels) throughout the cardiac cycle and offers the major advantage of detecting subtle cardiac dysfunction at an earlier stage than conventional functional echocardiography [16, 17]. There are tentative indications that prenatal myocardial deformation is decreased in fetuses with CoA, leading to the hypothesis that speckle-tracking echocardiography may aid in the prenatal diagnosis of CoA. However, previous studies yield conflicting results and are predominantly retrospective in design [18–24]. More importantly, a subset of them assessed structurally normal hearts as a control group rather than false-positive cases, limiting clinical applicability, since the key challenge for clinicians lies in differentiating true CoA cases from false-positives given the similarity in ultrasound features of these cases. No longitudinal study on this topic has been performed so far, while it is known that gestational age (GA) influences cardiac symmetry and myocardial strain parameters [25]. To reflect clinical practice, we conducted a longitudinal prospective study on fetuses with prenatal suspected CoA, based on asymmetrical cardiac biometry. We aimed at determining whether myocardial deformation analysis can aid in distinguishing true CoA cases from false-positives during the course of the pregnancy.

## Methods

This was a prospective observational cohort study performed at two tertiary referral centers for fetal cardiology (Leiden University Medical Center [LUMC] in the Netherlands and the German Heart Center Munich [GHCM] in Germany). Appropriate ethical standards have been followed. The study was reviewed and approved by the institutional Medical Ethics Review Boards of Leiden-Den Haag-Delft (NL66978.058.18/B19.060) and Technical University of Munich (555/19 S-KH). All participants gave written informed consent prior to inclusion.

### Patient Selection

All consecutive cases referred for prenatal suspicion of isolated CoA were recruited at our tertiary fetal cardiology services between January 2019 and October 2024 (LUMC) and between January 2019 and May 2022 (GHCM). Suspicion of CoA was defined as left-to-right cardiac disproportion with a smaller left side (ratio ≥1.5), either at ventricular level (right-to-left ventricular width ratio at end-diastole) or great arterial level (pulmonary-to-aortic valve ratio at end-systole or ductal-to-aortic isthmus ratio in the three-vessel-trachea view at end-systole). Cases with major extracardiac structural defects or additional cardiac anomalies (except for persistent left superior vena cava, restrictive or aneurysmatic foramen ovale, small ventricular septal defect or postnatal diagnosis of bicuspid aortic valve), were excluded, as were monochorionic twins. Among cases with a postnatal diagnosis of small left heart, only those without structural malformations of left-sided heart structures other than CoA (e.g., aortic valve stenosis, mitral valve dysplasia or stenosis) were included. Postnatal echocardiography and surgical reports defined the final diagnosis used for analysis. All neonates were admitted to the NICU after birth. Subjects were stratified into cases (true CoA) and controls (physiological right-sided dominance) according to their postnatal diagnosis and need for intervention: subjects requiring aortic arch surgery within the first year of life (coarctectomy with end-to-end anastomosis or aortic arch repair) were classified as true CoA cases, while those not requiring aortic arch surgery within the first life year were allocated to the false-positive control group.

### Data Collection

All subjects with prenatal suspicion of isolated CoA underwent a comprehensive ultrasound examination during each routine fetal echocardiographic consultation at our fetal cardiology services. At the LUMC, subjects were followed with serial fetal echocardiograms approximately every 4 weeks after initial diagnosis, whereas at the GHCM, only a single prenatal echocardiogram was typically performed. All cardiac examinations were conducted by experienced sonographers using the Canon Aplio I-series (i700-i900) ultrasound system (Canon Medical Systems Corporation, Tochigi, Japan) and were performed in accordance with the guidelines of the International Society of Ultrasound in Obstetrics and Gynecology (ISUOG) and American Society of Echocardiography [26–28]. The following cardiac dimensions were assessed on grayscale images: left and right ventricular length (at end-diastole), left and right ventricular width (at end-diastole), mitral and tricuspid valve diameters (at end-diastole), aortic and pulmonary valve annulus diameters (at end-systole) and aortic isthmus and arterial duct diameters (at end-systole). Respective Z-scores were computed according to the reference standards provided by Vigneswaran et al. [29] and Schneider et al. [30]. In addition, color Doppler recordings of the aortic valve and aortic arch were performed to assess flow velocities across the aortic valve and to determine the direction of blood flow through the aortic arch.

For speckle-tracking analysis, B-mode cine loops were recorded containing at least 4 cardiac cycles of the apical or basal cardiac four-chamber view, with the insonation angle 5°–45°, in absence of maternal and fetal movements (Fig. 1a, b). Settings were optimized by adjusting sector width, depth, gain and zoom box to achieve frame rates between 60 and 100 frames/s. The analysis of myocardial deformation was performed offline by a single observer (F.Z.), blinded for patient identification and neonatal outcome, using the 2D Wall Motion Tracking software of Canon Medical Systems. Recordings that did not meet the predefined acquisition criteria were excluded. If no analyzable recording was available for a given examination, that examination was excluded. Cases without any analyzable examination during follow-up were excluded from the study. For analysis, a cardiac cycle was manually selected by setting the time cursor between complete closure of the atrioventricular valves and just before the subsequent atrioventricular-valve closure. Endocardial markers were placed manually along the endocardial border in end-diastole, after which the software automatically traced the motion of the kernels throughout the cardiac cycle allowing for automatic calculation of global and segmental myocardial strain values. The following myocardial strain parameters were assessed separately for the right ventricle (RV) and left ventricle (LV): global longitudinal strain (GLS) and segmental longitudinal strain, global strain rate and ejection fraction. Mean values of 3 different cardiac cycles were used as definitive results. Within-fetus variability was assessed and defined as the agreement across the 3 separate cardiac cycles. Intraobserver agreement was assessed by repeating measurements on the same cardiac cycle from 15 randomly selected clips from different examinations with a minimum time interval of 2 months after original analysis. Interobserver variability for speckle-tracking analysis using Canon Medical Systems software has been previously demonstrated, with a good intraclass correlation coefficient of 0.87–0.91 [31].

**Fig. 1.**
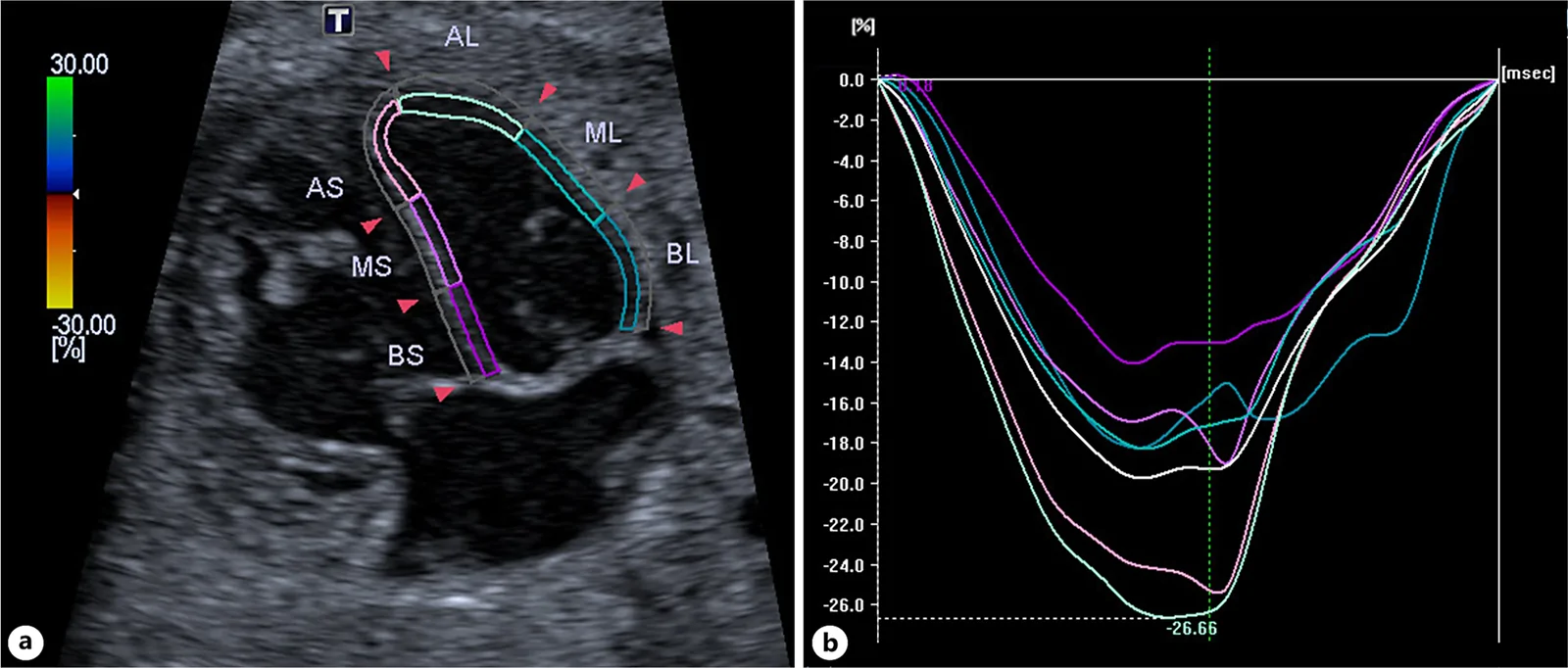
Schematic illustration of speckle-tracking echocardiography analysis in the cardiac four-chamber view of a fetus with suspected CoA. Representative example of automated myocardial border tracking of the left ventricle (**a**) and corresponding regional strain values over one cardiac cycle (**b**). BS, basal septal; MS, mid septal; AS, apical septal; AL, apical lateral; ML, mid lateral; BL, basal lateral.

Additional data on maternal and fetal characteristics, pregnancy course, and neonatal outcomes were retrieved from electronic patient records.

### Statistical Analysis

A power analysis based on previously reported data showing a 6.6% difference in left ventricular GLS [17] indicated that a minimum of 16 subjects per group (true CoA vs. false positives) was required to detect a significant difference (80% power, α = 0.05). All statistics were performed with SPSS Statistics 29.0 (IBM Corp., Armonk, NY, United States). Baseline characteristics are shown as frequencies (percentages), mean (±SD), or median (interquartile range). Independent *t*-tests, Chi-square tests and Fisher’s exact tests were applied as appropriate. Fetal cardiac dimensions and myocardial deformation were compared between true CoA and false-positives throughout gestation using linear mixed-effects models to account for repeated measurements, unequal time-intervals between examinations and variable numbers of examinations per subject. For speckle-tracking parameters, a random intercept and slope per subject were included, with GA at examination, group (true CoA vs. false-positives) and their interaction as covariates. Cardiac dimensions were modeled with only a random intercept, as the model with random slopes did not converge. Estimated means were reported as mean ± SE. A subgroup analysis of third-trimester examinations (≥33 weeks GA) was conducted using the Mann-Whitney U test. No adjustments were made for multiple comparisons, and results should be interpreted accordingly. The level of significance was set at *p* < 0.05. Intra-observer agreement and within-fetus variability were quantified using intraclass correlation coefficients.

## Results

### Case and Data Selection

During the study period, 49 subjects were prenatally referred for a suspected isolated CoA. We excluded 3 cases due to the presence of major additional cardiac anomalies identified on postnatal echocardiography (1 case with Scimitar syndrome and 2 with Shone complex) and 8 cases due to insufficient quality of the stored speckle-tracking clips. As a result, 38 cases were eligible for inclusion, of which 19 (50%) were true CoA cases, and 19 (50%) were false-positive cases. A total of 101 ultrasound examinations were performed on these subjects, of which 14 were not available for analysis due to a technical storage error and 7 due to insufficient quality of the stored speckle-tracking clips, leaving 80 examinations available for final analysis.

### Patient Characteristics

Baseline characteristics are depicted in Table 1. Relevant maternal and fetal baseline characteristics were comparable between true CoA and false-positive cases. True CoA cases were referred at earlier GA (21.0 vs. 30.3 weeks GA, *p* < 0.001) and consequently underwent more prenatal examinations (mean number of examinations 2.8 vs. 1.4, *p* = 0.002). Mean GA at study examination did not differ significantly between groups (28.7 vs. 30.7 weeks GA, *p* = 0.12). Cases with true CoA were more frequently diagnosed with a small ventricular septal defect (42.1% vs. 5.3%, *p* = 0.019) and a bicuspid aortic valve (57.9% vs. 0%, *p* < 0.001).

**Table 1.** Baseline Characteristics and Pregnancy Outcomes of Subjects Stratified by Postnatal Diagnosis

​	True CoA	False-positives	*p* value
	*n* = 19	*n* = 19	
Maternal age, years	33.5±5.5	32.1±3.6	0.36
Maternal BMI, kg/m^2^	25.1±4.8	25.1±5.5	0.99
GA at first presentation, weeks	21.0 (19.9–22.4)	30.3 (21.6–34.0)	**<0.001**
GA at research echocardiogram, weeks	28.7±5.3	30.7±5.8	0.12
Sex, male, *n* (%)	8 (42.1)	12/16 (63)	0.05
GA at birth	39.1 (38.0–39.6)	39.3 (38.4–40.0)	0.60
Birthweight, grams	3,438±574	3,246±820	0.41
Additional small cardiac findings, *n* (%)			
PLSVC	3 (15.8)	6 (31.6)	0.45
VSD (small)	8 (42.1)	1 (5.3)	**0.019**
Bicuspid aortic valve	11 (57.9)	0 (0)	**<0.001**
Restrictive or aneurysmatic foramen ovale	4 (21.1)	5 (26.3)	1.00
Presence of extracardiac anomaly^a^	1 (5.3)	3 (15.8)	0.60
Presence of fetal genetic abnormality^b^	2 (10.5)	2 (10.5)	1.00

Data given as mean ± SD, median (IQR), or *n* (%).

CoA, aortic coarctation; GA, gestational age; PLSVC, persistent left superior vena cava; VSD, ventricular septal defect.

^a^ Additional findings were unilateral hydronephrosis, single umbilical artery, echogenic bowels.

^b^ Genetic findings were Turner syndrome, Jacobsen syndrome, 10q deletion, reciprocal translocation.

### Cardiac Dimensions

Table 2 presents the mean fetal cardiac dimensions. True CoA cases exhibited smaller aortic valve and aortic isthmus diameters (mean aortic valve Z-score −3.2 vs. −1.6, *p* < 0.001; mean aortic isthmus Z-score −3.5 vs. −1.7, *p* = 0.001), while the diameters of the right-sided outflow tract were comparable between groups (mean pulmonary valve Z-score 3.0 vs. 2.6, *p* = 0.64; mean arterial duct Z-score 1.9 vs. 3.2, *p* = 0.61). Also, the mitral valve diameter was smaller in true CoA cases (mean Z-score −4.9 vs. −3.1, *p* < 0.001), while the tricuspid valve diameter did not differ (mean Z-score −0.8 vs. −1.1, *p* = 0.31). Mean Z-scores of left and right ventricular length and width were comparable between groups. In addition, true CoA cases were more frequently associated with the presence of an aortic shelf (36.8% vs. 5.3%, *p* = 0.04). Retrograde (diastolic or continuous) aortic arch flow occurred at comparable rates in the two groups (26.3% vs. 31.6%, *p* = 0.72).

**Table 2.** Cardiac Dimensions of Fetus With Suspected CoA, Stratified by Postnatal Diagnosis

​	True CoA	False-positives	*p* value	95% CI for mean difference
	*n* = 19	*n* = 19		
Outflow tracts				
AoV diameter, Z-score	−3.2±0.2	−1.6±0.3	**<0.001**	−2.32 to −0.87
PV diameter, Z-score	3.0±0.6	2.6±0.6	0.64	−1.32 to 2.11
PV/AoV ratio	2.3±0.1	1.8±0.1	**0.003**	0.18 to 0.80
Aortic isthmus diameter, Z-score	−3.5±0.4	−1.7±0.4	**0.001**	−2.83 to −0.74
Arterial duct diameter, Z-score	1.9±1.7	3.2±1.7	0.61	−6.14 to 3.65
Isthmus/arterial duct ratio	2.1±0.1	1.6±0.1	**0.005**	0.15 to 0.81
Four-chamber view				
LV length, Z-score	−1.3±0.2	−0.9±0.2	0.12	−0.98 to 0.11
RV length, Z-score	−1.1±0.5	−1.7±0.5	0.33	−0.71 to 2.06
LV width, Z-score	−2.1±0.2	−1.6±0.3	0.21	−1.31 to 0.29
RV width, Z-score	1.4±0.2	1.3±0.3	0.79	−0.65 to 0.85
RV/LV width ratio	1.9±0.1	1.7±0.1	0.21	−0.10 to 0.43
MV diameter, Z-score	−4.9±0.3	−3.2±0.3	**<0.001**	−2.65 to −0.90
TV diameter, Z-score	−0.8±0.2	−1.1±0.2	0.31	−0.29 to 0.89
TV/MV ratio	1.9±0.1	1.4±0.1	**<0.001**	0.25 to 0.66
Additional findings related to CoA, *n* (%)				
Aortic shelf	7 (36.8)	1 (5.3)	**0.04**	​
Retrograde aortic flow	5 (26.3)	6 (31.6)	0.72	​

Data given as mean ± SE or *n* (%). Mean values of cardiac dimensions were calculated using linear mixed models with a random intercept, including GA at examination, group (true CoA vs. false-positives), and their interaction as covariates. Statistically significant results are indicated in bold (*p* < 0.05).

AoV, aortic valve; CoA, aortic coarctation; LV, left ventricle; MV, mitral valve; PV, pulmonary valve; RV, right ventricle; TV, tricuspid valve.

### Myocardial Deformation

Myocardial deformation parameters are summarized in Table 3. Frame rates were comparable between true CoA and false-positive cases (81 vs. 74 fps, *p* = 0.31), as were mean fetal heart rate (148 vs. 151 bpm, *p* = 0.33) and cardiac cycle length (409 vs. 399 ms, *p* = 0.16). Mean GLS of the LV and RV did not differ significantly between groups (LV-GLS −20.1% vs. −20.5%, *p* = 0.57; RV-GLS −20.0% vs. −19.1%, *p* = 0.13). Also, segmental strain analysis of the ventricular walls revealed no differences (Online suppl. Table 1. For all online suppl. material, see https://doi.org/10.1159/fdt/acuag016). True CoA cases exhibited a significantly longer average time-to-peak for right ventricular strain compared with false-positive cases (221 vs. 200 ms, *p* = 0.028), while the left ventricle only showed a nonsignificant trend toward a longer time-to-peak strain (214 vs. 201 ms, *p* = 0.07). Diastolic and systolic strain rates of both ventricles were similar between groups (Table 3).

**Table 3.** Myocardial Strain Parameters in Fetus With Suspected CoA According to Postnatal Diagnosis

​	True CoA	False-positives	*p* value	95% CI for mean difference
	*n* = 19	*n* = 19		
Frame rate, fps	81±32	74±28	0.31	​
Fetal heart rate, bpm	148±12	151±12	0.33	​
Cardiac cycle length, ms	409±31	399±30	0.16	​
Left ventricle				
GLS – Average peak, %	−20.12±0.5	−20.54±0.6	0.57	(−1.10, 1.94)
GLS – Average time-to-peak, ms	214±4.7	201±5.9	0.07	(−1.35, 28.70)
Expressed as % of total cycle	52.1±1.1	50.1±1.5	0.48	(−2.43, 5.09)
GLSR – Average systolic peak, s^−1^	−2.1±0.1	−2.2±0.1	0.50	(−0.16, 0.33)
GLSR – Average time-to-syst. peak, ms	94±8.0	78±11.7	0.27	(−12.43, 44.06)
Expressed as % of total cycle	22.8±1.8	19.6±2.7	0.32	(−3.18, 9.61)
GLSR – Average diastolic peak, s^−1^	2.8±0.1	2.8±0.2	0.97	(−0.45, 0.47)
GLSR – Average time-to-diast. peak, ms	325±4.9	323±7.1	0.80	(−14.94, 19.28)
Expressed as % of total cycle	78.9±1.1	80.9±1.6	0.31	(−5.71, 1.83)
Ejection fraction	67.5±1.7	67.1±2.1	0.86	(−4.87, 5.78)
Right ventricle				
GLS – Average peak, %	−20.0±0.4	−19.1±0.48	0.13	(−2.10, 0.27)
GLS – Average time-to-peak, ms	221±5.7	200±7.4	**0.028**	(2.28, 39.48)
Expressed as % of total cycle	53.8±1.1	50.6±1.7	0.12	(−0.83, 7.14)
GLSR – Average systolic peak, s^−1^	−1.7±0.1	−1.7±0.1	0.53	(−0.13, 0.24)
GLSR – Average time-to-syst. peak, ms	93±7.0	79±10.6	0.30	(−11.88, 38.62)
Expressed as % of total cycle	22.6±1.7	19.6±2.6	0.34	(−3.22, 9.17)
GLSR – Average diastolic peak, s^−1^	2.9±0.1	2.5±0.2	0.09	(−0.06, 0.84)
GLSR – Average time-to-diast. peak, ms	341±5.5	328±7.7	0.18	(−6.12, 31.68)
Expressed as % of total cycle	83.0±1.0	82.4±1.4	0.71	(−2.78, 4.08)
Ejection fraction	50.7±1.5	50.2±1.8	0.85	(−4.23, 5.16)

Data given as mean ± SE. Mean values of strain parameters were calculated using linear mixed models with a random intercept and slope, including GA at examination, group (true CoA vs. false-positives), and their interaction as covariates. Statistically significant results are indicated in bold (*p* <0 .05).

bpm, beats per minute; CoA, aortic coarctation; fps, frames per second; GLS, global longitudinal strain; GLSR, global longitudinal strain rate; ms, milliseconds.

When comparing developmental changes over gestation, only the average time-to-peak for left ventricular strain differed significantly: the left ventricular time-to-peak strain increased with GA in false-positive cases whereas this effect was blunted in true CoA cases (rate of change in time-to-peak strain 1.96 vs. −0.55 ms/week, *p* = 0.032). All other strain parameters developed similarly over time.

A subgroup analysis was conducted using the final ultrasound examinations in the third trimester, when postnatal management decisions are typically made (i.e., whether to initiate prostaglandin therapy immediately after birth). At this pregnancy stage, there were also no statistical differences in left and right ventricular GLS and strain rates (Online suppl. Table 2).

### Intraobserver Agreement for Myocardial Deformation

Online supplementary Table 3 presents the intraobserver agreement and within-fetus variability. Intra-observer agreement was excellent for both left and right ventricular GLS, with intraclass correlation coefficients of 0.90 and 0.91 for single measures and 0.96 and 0.97 for averaged measures. The intraclass correlation coefficient for within-fetus variability was poor for single cycle measurements of GLS (LV 0.44, RV 0.52), reflecting high variability between cardiac cycles. When averaged over multiple cycles, within-fetus variability improved to a moderate-to-good level (LV 0.70, RV 0.76), indicating that the averaged strain values are reproducible and reliable. Segmental strain analysis showed comparable levels of intra-observer and within-fetus variability (Online suppl. Table 1).

### Postnatal Outcome

All neonates were delivered in a tertiary care center and admitted to the NICU after birth. Based on the prenatal conventional echocardiographic assessment by experienced clinicians, prostaglandin therapy was initiated directly postpartum in 86.7% of true CoA cases and in 20.0% of false-positive cases (*p* < 0.001). Subjects not directly receiving prostaglandin therapy were closely monitored with strict echocardiographic surveillance until ductal closure to detect any signs of CoA. In true CoA cases, aortic repair surgery was performed at a median of 16 days postpartum (range 8–49 days). The false-positive cases were discharged without neonatal intervention in the first life year.

## Discussion

This study prospectively assessed myocardial deformation throughout gestation in fetuses with a prenatal suspicion of isolated CoA, comparing those postnatally confirmed as true CoA with those identified as false-positives. Key findings were that true CoA cases exhibited significantly smaller left-sided cardiac diameters, but did not differ from false-positive cases in myocardial deformation indices. Global longitudinal strain, strain rate, and segmental strain values were comparable between the groups throughout pregnancy. Only right ventricular time-to-peak strain was modestly prolonged in true CoA cases, while the rate of change during gestation of left ventricular time-to-peak strain appeared blunted compared with false-positives. These findings indicate that myocardial deformation analysis provides no additional diagnostic value for the prenatal differentiation of true CoA from false-positive cases.

This study differs from previous reports by demonstrating no clinically relevant differences in myocardial deformation between true CoA and false-positive cases, challenging earlier recommendations that altered strain patterns could distinguish the two groups. Notably, prior studies on this topic are limited and show substantial variability, with reported strain abnormalities ranging from isolated reduction in left ventricular strain [18, 22–24], to exclusive right ventricular strain decrease [20] and, in one study, biventricular involvement [21]. These discrepancies are likely attributable to considerable methodological and clinical heterogeneity among the studies. The most notable difference with the current study is the comparison with normal fetuses rather than false-positive cases in most previous papers [23], a methodological design that not only overestimates the diagnostic capacity in CoA cases, but also does not mirror the clinical challenge in which differentiation between true CoA and false-positive cases must be made prenatally [18, 23]. In addition, several studies included fetuses with concomitant cardiac anomalies – such as partial anomalous pulmonary venous return, interrupted aortic arch, cardiomyopathy, or mitral valve anomalies – which may independently alter myocardial deformation and therefore limit the applicability of their findings to isolated CoA [19, 21]. Other methodological heterogeneities further complicates interpretation of the literature: prior studies varied markedly in frame rates, ultrasound equipment, speckle-tracking software (each with distinct algorithms) and insonation angles of the four-chamber view [18–24], all of which are known to affect strain quantification [31]. A recent systematic review by Van den Wildenberg et al. pooled data from all aforementioned studies and reported a 4.5% reduction in left ventricular strain without a difference in right ventricular strain between true CoA cases and controls [32]. However, given that the review is constrained by the methodological inconsistencies inherent to the included studies, it is unlikely that this modest GLS reduction is a reproducible or clinically meaningful predictor of postnatal outcome. The present study addresses previous limitations by focusing on fetuses with isolated CoA and analyzing myocardial deformation longitudinally in a prospective cohort, directly comparing confirmed CoA with false-positive cases – the most clinically relevant distinction in prenatal setting. Although true CoA cases showed minor delays in ventricular time-to-peak strain and blunted gestational increase in left ventricular time-to-peak strain, these nuanced changes, while detectable in a research setting, seem insufficient to serve as reliable clinical biomarkers for distinguishing true CoA from false-positive cases. Moreover, strain measurements are inherently sensitive to image acquisition, including fetal position, cardiac orientation, and insonation angle. Although we applied strict acquisition criteria, including a predefined insonation range of 5–45°, 14% of examinations were excluded because of inadequate image quality. Still, residual technical variability cannot be excluded. This inherent limitation further constrains the clinical implementation of fetal speckle-tracking echocardiography in prenatal diagnosis.

The use of speckle-tracking analysis to assess fetuses with suspected CoA was based on the hypothesis that true CoA alters ventricular loading – in particular left ventricular afterload – differently due to actual aortic obstruction, compared with the physiological right-sided dominant hemodynamic conditions in false-positive cases. In our cohort, however, myocardial deformation did not differ between the groups and even remained within reported normal reference ranges [25], also when assessed longitudinally throughout gestation, suggesting that the hypothesized load increase in CoA does not occur in utero or could not be objectified by speckle-tracking. The findings in this study support the concept that the characteristic cardiac asymmetry in CoA primarily reflects relative underfilling of the left heart, rather than compensatory remodeling or intrinsic myocardial dysfunction due to increased loading conditions. In the normal fetal circulation, systemic output can bypass the aortic isthmus through the right heart via the open foramen ovale and arterial duct. We hypothesize that these fetal shunts effectively preserve left ventricular loading conditions by facilitating blood flow redistribution in case of aortic narrowing, as corroborated by findings of a recent computational modeling study demonstrating that aortic isthmus obstruction is accompanied by a compensatory increased right-sided output and ductal flow, maintaining physiological pressures and wall shear stresses [33]. The nuanced prolongation of right ventricular time-to-peak strain observed in true CoA in this study further supports this hypothesis, as it may reflect a slightly increased right ventricular workload or altered contraction timing secondary to preferential right-sided output. Yet, this increase in time-to-peak strain is subtle and appears insufficient for clinical application. Notably, our prior study in fetal aortic stenosis demonstrated that severe myocardial strain reduction correlated with stenosis severity and the amount of pathological fibroelastosis and cardiomyocyte disorganization, suggesting that evident fetal myocardial strain impairment predominantly emerges under more extreme altered loading conditions [17]. False-positive cases may share hemodynamic features with true CoA – such as augmented right-sided output and ductal flow – which likely arise from normal physiological variability rather than structural obstruction. This interpretation is supported by the occurrence of retrograde ductal flow in some false-positive cases in the current study, reflecting physiological redistribution of blood flow across the fetal shunts (e.g., right-sided dominance) in the absence of aortic narrowing [34]. As a result of these shared hemodynamic features, myocardial loading conditions and deformation patterns substantially overlap between groups, further limiting the discriminative ability of fetal strain analysis in this diagnostic setting.

Importantly, the absence of detectable differences does not exclude a potential effect of CoA on myocardial deformation in more severe forms of the disease. CoA represents a heterogeneous spectrum ranging from discrete narrowing to more extensive tubular hypoplasia. Any potential myocardial involvement may therefore be confined to cases with more extensive anatomical obstruction. However, this could not be evaluated in the present study due to the limited number of true CoA cases and the inherent difficulty in defining disease severity in CoA, which is influenced by anatomical extent, timing of clinical presentation, and timing of intervention.

In the current study, prostaglandin therapy was initiated immediately after birth in 87% of true CoA cases compared with only 20% of false-positive cases, based solely on conventional echocardiographic assessment by experienced clinicians. Although this corresponds to strong positive and negative predictive values of 81% and 86%, all neonates with a prenatal suspicion of CoA were delivered in tertiary care and routinely admitted to the NICU. This persistent precaution reflects current clinical reality: no anatomical or functional imaging marker currently provides sufficient diagnostic certainty to safely triage perinatal management. Even with promising results of fetal MRI studies [11], clinicians remain justifiably cautious, as missing a true CoA could have serious consequences if appropriate postnatal care is delayed [2]. To our opinion, the field must therefore acknowledge that improving prenatal prediction requires approaches beyond imaging alone, such as artificial intelligence, genomics, or advanced transcriptomic profiling [35–37].

### Strengths and Limitations

The current study most accurately reflects the clinical setting of prenatally suspected fetal CoA due to its prospective, longitudinal design comparing true CoA cases with false-positives. Nevertheless, despite an a priori power calculation, the absolute number of cases with prenatally suspected CoA remains relatively small. CoA encompasses a heterogeneous spectrum of severity, ranging from focal shelves to more extensive tubular hypoplasia. The influence of disease severity on our findings cannot be fully excluded, and a meaningful subgroup analysis was not feasible because of a lack of power for subdivision. However, severe cases are typically associated with more pronounced cardiac asymmetry or narrowing of the aortic arch, resulting in less frequent diagnostic uncertainty. The clinically relevant challenge therefore lies mostly in fetuses with mild or borderline findings, in which myocardial deformation does not appear affected. Additionally, we did not include a control group of healthy fetuses, which could have provided further insight into the underlying pathophysiology of cardiac asymmetry.

## Conclusion

Fetal myocardial deformation parameters derived from speckle-tracking echocardiography did not differ between true CoA and false-positive cases throughout gestation. These findings indicate that myocardial deformation analysis does not provide additional predictive value for postnatal outcome in fetuses with a prenatal suspicion of isolated CoA. As this prospective study most accurately reflects the clinical setting, we warrant that further research in this regard is of limited utility. It should be acknowledged that current imaging modalities cannot provide sufficient diagnostic certainty to safely triage perinatal management. Future improvement in prenatal prediction will likely require approaches beyond imaging, such as artificial intelligence or molecular biomarkers.

## Acknowledgments

We thank Canon Medical Systems, who kindly provided technical support in the software for this study.

## Statement of Ethics

The study was reviewed and approved by the institutional Medical Ethics Review Boards of Leiden-Den Haag-Delft (NL66978.058.18) and Technical University of Munich (555/19 S-KH). All participants gave written informed consent prior to inclusion.

## Conflict of Interest Statement

The authors have no conflicts of interest to declare.

## Funding Sources

This study was not supported by any sponsor or funder.

## Author Contributions

All listed authors have contributed to the manuscript substantially and have agreed to the final submitted version. Conceptualization and design: F.Z., M.C.H., and A.D.J.T.H.; acquisition: F.Z., J.T.-R., R.M.S., R.O., and A.W.-G.; analysis: F.Z.; interpretation of data: F.Z., M.C.H., A.D.J.T.H, R.O., and A.W.-G.

## Data Availability

The data that support the findings of this study are not publicly available due to privacy reasons but are available from the corresponding author upon request.
